# What is a clinical pathway? Development of a definition to inform the debate

**DOI:** 10.1186/1741-7015-8-31

**Published:** 2010-05-27

**Authors:** Leigh Kinsman, Thomas Rotter, Erica James, Pamela Snow, Jon Willis

**Affiliations:** 1 School of Rural Health, Monash University, Bendigo, Victoria, Australia; 2 Department of Public Health, University of Dresden, Dresden, Germany; 3 Centre for Health Research & Psycho-oncology (CHeRP), The University of Newcastle, Newcastle, New South Wales, Australia; 4 School of Psychology and Psychiatry, Monash University, Bendigo, Victoria, Australia; 5 School of Public Health, La Trobe University, Bendigo, Victoria, Australia

## Abstract

**Background:**

Clinical pathways are tools used to guide evidence-based healthcare that have been implemented internationally since the 1980s. However, there is widespread lack of agreement on the impact of clinical pathways on hospital resources and patient outcomes. This can be partially attributed to the confusion for both researchers and healthcare workers regarding what constitutes a clinical pathway. This paper describes efforts made by a team of Cochrane Review authors to develop criteria to assist in the objective identification of clinical pathway studies from the literature.

**Methods:**

We undertook a four-stage process aiming to develop criteria to define a clinical pathway: (1) identify publications exploring the definition of a clinical pathway; (2) derive draft criteria; (3) pilot test the criteria; and (4) modify criteria to maximise agreement between review authors.

**Results:**

Previous literature and liaison with the European Pathways Association resulted in five criteria being used to define a clinical pathway: (1) the intervention was a structured multidisciplinary plan of care; (2) the intervention was used to translate guidelines or evidence into local structures; (3) the intervention detailed the steps in a course of treatment or care in a plan, pathway, algorithm, guideline, protocol or other 'inventory of actions'; (4) the intervention had timeframes or criteria-based progression; and (5) the intervention aimed to standardise care for a specific clinical problem, procedure or episode of healthcare in a specific population. After pilot testing it was decided that if an intervention met the first criteria (a structured multidisciplinary plan of care) plus three out of the other four criteria then it was included as a clinical pathway for the purposes of this review. In all, 27 studies were included in the final review. The authors of the included studies referred to these interventions as 'clinical pathways', 'protocols', 'care model', 'care map', 'multidisciplinary care', evidence-based care' and 'guideline'.

**Conclusions:**

The criteria used for the identification of relevant studies for this Cochrane Review can be used as a foundation for the development of a standardised, internationally accepted definition of a clinical pathway.

## Background

In 2003, it was reported that clinical pathways had been implemented in more than 80% of hospitals in the USA. This represents an enormous resource commitment both in the development of pathways, the training of staff, and in the ongoing implementation of pathways in the hospital setting. In this era of evidence informed practice, it is therefore problematic that individual studies of the impact of clinical pathways are varied and contradictory [1] and that there is still no standardised definition of what a 'clinical pathway' actually constitutes. In fact, a recent literature review identified 84 different terms that may mean a clinical pathway. These included (amongst others) care map, care pathway, critical pathway, integrated care pathway, protocol and guideline [2].

This lack of a uniformly accepted definition of what constitutes a clinical pathway impacts on capacity to empirically test the evidence base and compromises planning, resourcing, development and implementation of clinical pathways. A lack of consensus regarding research outcomes is not surprising given the lack of agreement regarding what defines a clinical pathway.

Our team of researchers faced this issue when devising the protocol for a Cochrane systematic review of the impact of clinical pathways in hospitals [3]. The development of minimum criteria to define a clinical pathway was required to ensure that appropriate studies were sourced and included in the review. The aim of this paper is to describe the developmental process undertaken and the resulting criteria so that other researchers who are developing and evaluating clinical pathways or attempting to synthesise the existing literature can consider the usefulness of these criteria for their studies.

## Method

We undertook a four-stage process aiming to develop evidence-informed and practical criteria to define a clinical pathway. The four stages were: (1) identify publications exploring the scope and definition of clinical pathways (or similar terms); (2) synthesise previously suggested components and derive draft criteria for testing; (3) pilot test the level of agreement between review authors when applying criteria to identified studies; and (4) modify criteria to maximise agreement between review authors.

## Results

### Literature

A search of electronic databases and communication with the European Pathways Association revealed three 'sentinel' articles that described the characteristics of a clinical pathway: Campbell *et al*., De Bleser *et al*. and Vanhaecht *et al*. [2,4,5]. De Bleser *et al*. surveyed the multiple terms used to describe a clinical pathway via a comprehensive review of the literature and derived key characteristics in attempting to address international confusion regarding the definition of a clinical pathway [2]. Campbell *et al*. described clinical pathways in the context of their relationship to clinical guidelines [4], whilst Vanhaecht *et al*. summarised previous studies to determine whether their review of audit tools elicited common characteristics of clinical pathways [5]. A summary of the characteristics identified by these studies is provided in Table 1.

**Table 1:** Characteristics of clinical pathways derived from sentinel articles

**De Bleser *et al*. **[2]	**Campbell *et al*. **[4]	**Vanhaecht *et al*. **[5]
Guides care management for a well defined group of patients for a well defined period of time	Structured multidisciplinary care plan	Facilitate variance management
States goals and key elements of care based on evidence and best practice	Detail essential steps in care of patients with a specific clinical problem	Support multidisciplinary care
Sequences the actions of a multidisciplinary team	Facilitate translation of national guidelines into local protocols	Support evidence-based clinical practice
Allow documenting, monitoring and evaluating of variances	Help communication with patients by providing a clearly written summary of care	

### Criteria

The following five criteria were derived from the three sentinel articles mentioned above: (1) the intervention was a structured multidisciplinary plan of care; (2) the intervention was used to channel the translation of guidelines or evidence into local structures; (3) the intervention detailed the steps in a course of treatment or care in a plan, pathway, algorithm, guideline, protocol or other 'inventory of actions'; (4) the intervention had timeframes or criteria-based progression (that is, steps were taken if designated criteria were met); and (5) the intervention aimed to standardise care for a specific clinical problem, procedure or episode of healthcare in a specific population.

### Level of agreement and final criteria

These criteria were tested by three of the team on five papers. If the intervention described in the paper met all five criteria then it was considered a clinical pathway. This resulted in agreement between reviewers on only two out of the five papers. It was apparent that the main obstacle to agreement on all five criteria was the poor reporting of the intervention. To address this issue the criteria were unchanged but their relative importance was adjusted. An intervention was defined as a clinical pathway if it was a structured multidisciplinary plan of care and at least three of the remaining four criteria were met (that is, it met the first criteria and any three of the remaining four). This amended schedule of essential criteria was then tested by applying them to a further five papers. Following the application of the amended schedule of criteria, there was 100% agreement between the three review authors regarding whether an intervention was a clinical pathway. This schedule was then adopted by the review group and applied to studies identified by the search strategy for the systematic review.

### Application of criteria

The weighted criteria were applied to 260 full text articles. Two review authors independently screened articles to assess which studies met the criteria for a clinical pathway and the Cochrane methodological quality criteria. The Cochrane methodological criteria relates to minimum quality standards for quantitative studies using randomised controlled trial, controlled clinical trial, controlled before and after and interrupted time series study designs [6]. Unresolved disagreements on inclusion were referred to a third review author. In all, 63 papers were excluded as they did not meet the criteria defining a clinical pathway. Only two studies needed to be referred to a third review author because of disagreement on a study meeting the minimum definition criteria.

A total of 27 studies were included on both definition and methodological criteria in the final review. In all, 14 studies termed their intervention a clinical pathway whilst 8 others referred to their intervention as a 'protocol'. No other term was used more than once. The remaining terms used for what we included as a clinical pathway were 'care model', 'care map', 'multidisciplinary care', 'evidence-based care' and 'guideline'.

## Discussion

The criteria developed in this study were derived from a critical analysis of existing literature and pilot testing to ensure the practicality of their use. Though broadly inclusive, they were designed to have clear parameters in order to allow development of objective inclusion criteria. The search strategy used in the Cochrane review was also deliberately inclusive so that all possible literature was screened for inclusion. Subsequently the individual empirical studies included in the review often included interventions described by the primary authors as something other than a clinical pathway. This was borne out by the fact that only 14 out of the final 27 included studies used the term 'clinical pathway' to describe their intervention. The existence of interventions called something other than clinical pathways that met the same criteria is evidence of confusion around what constitutes a clinical pathway. This strongly suggests that the development of an acceptable, objective and applicable definition would overcome much confusion about what constitutes a clinical pathway. The criteria described here provide a sound basis for further discussion amongst researchers and clinicians with the desire being to move towards an internationally acceptable definition.

There was a high level of agreement between authors when criteria were applied, as shown by the fact only 2 studies were referred to a third author due to disagreement, whilst there was agreement on the exclusion of 63 other studies. Therefore, these criteria demonstrate strong potential to be clear and objective enough for broad agreement and use by publishers, researchers, clinicians and anyone else interested in the investigation of clinical pathways in healthcare.

The criteria developed for this review could be used as a framework for describing interventions that may be considered a clinical pathway. This would strengthen the capacity of clinicians, researchers, educators and policymakers to locate and apply relevant evidence regarding the use of clinical pathways.

## Conclusions

This paper described the developmental process undertaken to devise criteria to define a clinical pathway. Researchers who are developing and evaluating clinical pathways or attempting to synthesise the existing literature should consider the usefulness of these criteria for their studies. It is also recommended that these criteria be used as a basis for ongoing development of a standardised, internationally accepted definition of a clinical pathway.

## Competing interests

The authors declare that they have no competing interests.

## Authors' contributions

LK, TR and EJ participated in development and testing of criteria, and to the preparation of the manuscript. PS and JW contributed to study design and writing of the manuscript.

## Pre-publication history

The pre-publication history for this paper can be accessed here:

http://www.biomedcentral.com/1741-7015/8/31/prepub
